# Real-Time Monitoring of Microbial Contamination and Stress Biomarkers with Liquid Crystal-Based Immunosensors for Food Safety Assessment

**DOI:** 10.3390/bios16010059

**Published:** 2026-01-13

**Authors:** Maria Simone Soares, Andreia C. M. Rodrigues, Sílvia. F. S. Pires, Amadeu M. V. M. Soares, Ana P. L. Costa, Jan Nedoma, Pedro L. Almeida, Nuno Santos, Carlos Marques

**Affiliations:** 1 CICECO–Aveiro Institute of Materials & Physics Department, University of Aveiro, Campus Universitário de Santiago, 3810-193 Aveiro, Portugal; msimone.fsoares@ua.pt; 2 I3N & Physics Department, University of Aveiro, Campus Universitário de Santiago, 3810-193 Aveiro, Portugal; nfsantos@ua.pt; 3 CESAM—Centre for Environmental and Marine Studies and Department of Biology, University of Aveiro, 3810-193 Aveiro, Portugal; rodrigues.a@ua.pt (A.C.M.R.); silviapires1@ua.pt (S.F.S.P.); asoares@ua.pt (A.M.V.M.S.); 4 SEAentia-Parque Tecnológico de Cantanhede, Núcleo 04 Lote 2, 3060-197 Cantanhede, Portugal; anaplcosta@seaentia.pt; 5 Department of Telecommunications, VSB–Technical University of Ostrava, 70800 Ostrava, Czech Republic; jan.nedoma@vsb.cz; 6 I3N–CENIMAT, School of Sciences and Technology, NOVA University of Lisbon, 2829-516 Caparica, Portugal; pedro.almeida@isel.pt; 7 UnIRE, ISEL, Polytechnic University of Lisbon, 1959-007 Lisbon, Portugal; 8 Department of Physics, VSB–Technical University of Ostrava, 70800 Ostrava, Czech Republic

**Keywords:** aquaculture, biosensor, cortisol, depuration, *Escherichia coli* (*E. coli*), liquid crystal (LC), recirculating aquaculture system (RAS)

## Abstract

Aquaculture is a crucial global food production sector that faces challenges in water quality management, food safety, and stress-related health concerns in aquatic species. Cortisol, a key stress biomarker in fish, and *Escherichia coli* (*E. coli*) contamination in bivalve mollusks are critical indicators that require sensitive and real-time detection methods. Liquid crystal (LC)-based immunosensors have emerged as a promising solution for detecting biological analytes due to their high sensitivity, rapid response, and label-free optical detection capabilities. Therefore, this study explores the development and application of LC-based immunosensors for the detection of cortisol in artificial and real recirculating aquaculture system (RAS) samples, as well as *E. coli* in real contaminated water and clam samples during the depuration processes of bivalve mollusks. The biosensors exhibited the capacity to detect cortisol with a response time in seconds and a limit of detection (LOD) of 0.1 ng/mL. Furthermore, they demonstrated specificity to cortisol when tested against different interfering substances, including testosterone, glucose, and cholesterol. Furthermore, it was possible to correlate cortisol concentrations in different filtration stages and track *E. coli* contamination during depuration. The results confirm the feasibility of LC-based immunosensors as a user-friendly, portable, and efficient diagnostic tool for aquaculture applications.

## 1. Introduction

Aquaculture has emerged as a crucial global food production sector, providing a significant portion of the world’s seafood consumed worldwide. However, this industry faces several challenges, including water quality issues, disease outbreaks, and stress-related health problems in aquatic species [1]. Recirculating aquaculture systems (RASs) have been developed as a sustainable alternative to traditional aquaculture by reducing environmental impacts and offering efficient water reuse and better control over environmental conditions. Despite this progress, challenges persist in managing water quality and reducing stress on fish [2]. Similarly, estuarine ecosystems, which often host aquaculture operations, are particularly susceptible to contamination by harmful bacteria like *Escherichia coli* (*E. coli*). This raises concerns about food safety, as these environments are natural habitats for bivalve mollusks that are consumed in large quantities [3].

Stress is a major health concern that affects a significant portion of the population, regardless of age, environment, socioeconomic status, or other factors [4]. Cortisol is a stress hormone that plays a crucial role in critical sectors, such as food quality and production, particularly in aquaculture, where it serves as a key indicator of fish well-being. Prolonged elevated levels impair growth, immunity, and productivity, making stress monitoring essential [5,6]. In the RAS, tracking cortisol helps identify stressors like poor water quality or overcrowding, enabling timely intervention [7,8,9,10]. Traditional cortisol measurements are invasive, but studies have shown that fish release cortisol into the surrounding water, allowing non-invasive monitoring. In particular, European sea bass reared at high stocking densities exhibit significantly greater cortisol concentrations in water (≈3.084 ng/mL) than those maintained at low densities (≈0.007 ng/mL) [11]. More recent research further confirms that cortisol detection in water samples can improve fish welfare and aquaculture efficiency by reducing stress-related issues and, consequently, enhancing productivity [12]. A 2014 study measured cortisol concentrations in RAS water across several commercial farms. The species included Atlantic salmon (*Salmo salar*), rainbow trout (*Oncorhynchus mykiss*), European sole (*Pleuronectes platessa*), European eel (*Anguilla anguilla*), sturgeon (*Acipenser* spp.), and turbot (*Scophthalmus maximus*), with cortisol levels ranging from 0.0038 to 0.2170 ng/mL [13]. A separate 2017 study on Nile tilapia (*Oreochromis niloticus*) reported cortisol concentrations ranging from 0.0010 to 0.0051 ng/mL, with an increase of 30% after handling [14]. Species, fish density, water quality, and handling practices influence these levels. These findings underscore the importance of monitoring waterborne stress indicators in RAS. In these systems, the physical components of the water treatment loop can influence the concentration and persistence of waterborne cortisol. Specifically, biofilters host complex and active microbial communities that may interact with steroid hormones in the water. Processes such as microbial degradation, enzymatic transformation, and adsorption onto biofilms or filter media can reduce cortisol levels and alter its effective half-life within the system. Additional steps, like ozonation, can further improve hormone removal. These mechanisms can create spatial differences in cortisol concentrations at various filtration stages of the RAS, which is important to consider when analyzing waterborne cortisol data and conducting comparative sampling [13,15].

*E. coli* contamination in aquaculture, especially in bivalve mollusks, is a major food safety concern, requiring effective monitoring and depuration. As filter feeders, mollusks accumulate contaminants, making them vulnerable to *E. coli* from sewage, agricultural runoff, and stormwater [16,17,18]. Certain bacterial strains, like *E. coli* O157:H7, produce harmful toxins, while non-pathogenic strains indicate possible contamination with other pathogens [16,19]. Consuming contaminated mollusks can cause severe illness, highlighting the need for strict safety measures [20]. Depuration helps reduce microbial loads, and regulatory agencies enforce microbiological standards. However, the official international reference approach for evaluating bivalve mollusk contamination with *E. coli* using the most probable number (MPN) is time-consuming, taking 2 days to obtain results [16,21].

Ongoing research and technological advancements, such as user-friendly tools like biosensors for real-time cortisol and *E. coli* detection, are essential for further improving monitoring and purification techniques in the seafood industry.

Liquid crystal-based sensors are gaining attention due to their unique optical properties, high sensitivity, and versatility in detecting various analytes [22]. These sensors respond to external stimuli, such as biomolecular interactions, chemicals, or environmental changes, by changing the orientation of liquid crystal molecules. This molecular reorientation can be triggered by surface-anchored liquid crystal (LC) changes, electric or magnetic fields, or chemical stimuli and is detectable via polarized light microscopy, spectroscopy, or electrical methods. Specifically, target analytes can disrupt LC alignment by immobilizing biomolecules like antibodies or aptamers on the sensor surface, causing measurable optical shifts that can be easily detected using polarized light, making these sensors a promising tool for real-time and label-free detection of biological target analytes [23,24,25]. These changes can be observed using polarized optical microscopy (POM) or portable optical devices. Therefore, these biosensors offer significant advantages over traditional cortisol and *E. coli* biosensors, including label-free detection, real-time visualization, and cost-effectiveness. Competing biosensors, such as electrochemical, optical, piezoelectric, and molecular methods (PCR, culture-based), face challenges like complex instrumentation, lengthy processing times, environmental interference, and high costs. Unlike these alternatives, LC-based biosensors provide rapid, sensitive, and non-invasive detection, making them ideal for application in several areas, including medical diagnostics, food safety, and environmental monitoring [26,27,28].

In the literature, it is challenging to find many works on LC-based sensors for *E. coli* and, in particular, cortisol detection. Different approaches using liquid crystals can be applied for *E. coli* detection. An earlier work reported by H. Xu et al. demonstrated a method for detecting *E. coli* using the nematic liquid crystal 4-Cyano-4′-pentylbiphenyl (5CB). When *E. coli* cells were introduced onto 5CB-treated surfaces, distinct changes in LC orientation were observed under a polarizing optical microscope, indicating the potential of LCs for bacterial detection [29]. M. Liu et al. later developed an LC-based aptasensor for ultrasensitive *E. coli* detection. The binding of bacteria to aptamers disrupted LC alignment, causing observable optical variations and achieving an ultralow limit of detection (LOD) of 27 CFUs/mL, with potential applications in environmental and food safety monitoring [30]. In our previous work, we developed and validated an LC-based optical immunosensor for the rapid and sensitive detection of *E. coli*. The sensor operates by leveraging the alignment disturbance of nematic LC molecules upon interaction with the whole *E. coli* cells. The detection mechanism relies on the binding of *E. coli* to the specific anti-*E. coli* antibodies immobilized on the sensor surface, leading to observable optical changes under crossed linear polarizers. This immunosensor achieved an LOD of 2.8 CFUs/mL, outperforming many existing LC-based and traditional biosensors [31]. Regarding cortisol sensing, Tsung-Keng Chang et al. introduced an innovative method to enhance the sensitivity of LC-based biosensors by utilizing a marginally tilted alignment of LC molecules, eliminating the need for external electric fields. Using E7 and 5CB LCs between modified glass substrates, they detected BSA and cortisol with LODs of 2.5 × 10^−5^ ng/mL and 3 × 10^−3^ ng/mL, respectively, based on optical textures observed under a polarized microscope [32]. Saba Mostajabodavati et al. reported a portable, label-free LC aptasensor for cortisol detection in saliva and sweat, enhanced by smartphone integration and image-based machine learning analysis. The sensor detects LC alignment changes triggered by aptamer–cortisol interactions, with a response time under 8 min, a linear range of 1–150 ng/mL, and an LOD of 0.414 ng/mL [33]. To date, no other work has been reported in the literature regarding LC-based sensors for *E. coli* and, particularly, for cortisol detection.

**Figure 1:**
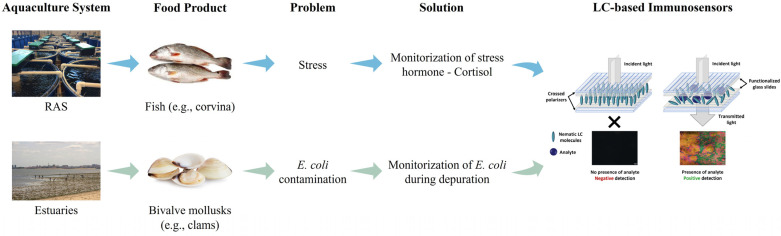
Summary outline of our work objective.

The present study focuses on addressing the problems found in the aquaculture industry through the development of LC-based immunosensors as a portable sensing platform. These sensors were designed to detect cortisol and *E. coli* with high sensitivity and specificity. In our previous work [31], these immunosensors were developed and only tested for *E. coli* concentrations obtained by culture media in the laboratory. Here, the present work involves testing these immunosensors in real scenarios, including RAS environments (with corvina—*Argyrosomus regius* meagre) for cortisol sensing, in contrast to the aforementioned works of Tsung-Keng Chang et al. [32] and Saba Mostajabodavati et al. [33], and depuration settings (with clams, specifically *Venerupis corrugata*) to complement the previous work on *E. coli* detection (Figure 1). To evaluate their performance, this study demonstrates the sensors’ effectiveness in monitoring cortisol in water samples from RAS at different stages of filtration and in assessing *E. coli* concentrations in contaminated water and bivalve mollusks during depuration processes. Importantly, portability is achieved by integrating the LC-based immunosensors with a compact optical readout prototype that replaces conventional POM, enabling rapid, on-site signal acquisition without complex instrumentation. The LC-based immunosensors developed for cortisol detection were analyzed both by POM and using the prototype for comparative evaluation. The results highlight the potential of these immunosensors for improving aquaculture management, ensuring food safety, and supporting the health of aquatic species and ecosystems. This work represents a step forward in the development of portable, user-friendly diagnostic tools that can be applied in practical aquaculture settings.

## 2. Materials and Methods

### 2.1. Reagents

Hydrogen peroxide (H_2_O_2_, 30% *v*/*v*) was purchased from Merck, Darmstadt, Germany. Sulfuric acid (H_2_SO_4_, 95–97%) was obtained from Fluka, Buchs, Switzerland. Bovine serum albumin (BSA, pH = 7.0) was obtained from ITW Reagents, Monza, Italy. Phosphate-buffered saline (PBS) tablets (pH = 7.4, 10 mM) and ethanol absolute (≥99.8%) were acquired from Fisher Bioreagents, Waltham, MA, USA. *N*-hydroxysuccinimide (NHS, 98%), *N*-(3-dimethylaminopropyl)-*N*′-ethylcarbodiimide hydrochloride (EDC, ≥98%), (3-aminopropyl)triethoxysilane (APTES, 99%), hydrocortisone (cortisol, ≥98%), 17α-methyltestosterone (testosterone, ≥97%), cholesterol (≥99%), and D-(+)-glucose (≥99.5%) were purchased from Sigma-Aldrich, Taufkirchen, Germany. *N*,*N*-dimethyl-*N*-octadecyl-3-aminopropyl trimethoxysilyl chloride (DMOAP, 60% in methanol) was purchased from ThermoFisher Scientific, Basel, Switzerland. Cortisol polyclonal antibody (5 mg/mL) and *E. coli* serotype O/K polyclonal antibody (4–5 mg/mL) were purchased from Invitrogen, Waltham, MA, USA. Deionized (DI) water was obtained from the Milli-Q water purification system. The epoxy glue (Araldite) was obtained from Ceys, Barcelona, Spain. The nematic LC 4-cyano-4′-pentylbiphenyl (5CB), supplied by Merck (Darmstadt, Germany) under the product name K15, was used as received without additional purification. Standard 26 × 76 mm microscope slides, cut into 26 × 10 mm pieces for sensor fabrication, were purchased from ThermoFisher Scientific, Basel, Switzerland.

### 2.2. Sensor Preparation: Functionalization, Assembly, Detection Mechanism, and Characterization

**Figure 2:**
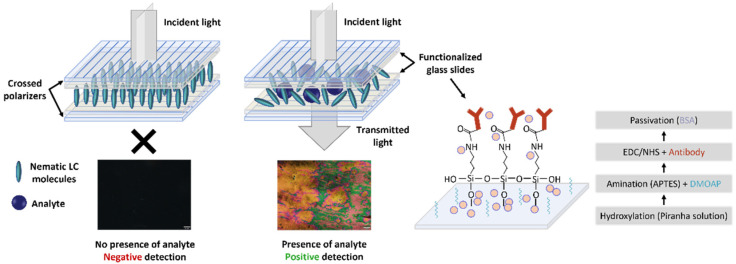
Schematic pictures of sensor assembly, detection mechanism, and glass slide’s functionalization. The colors obtained in the positive detection POM photographs are due to the chaotic rotation of the incident light polarization (between cross polarizers), promoted by the liquid crystal molecules orientation distortion imposed by the presence of the analyte.

To develop a specific biosensor for cortisol and another for *E. coli*, the glass slides, with 26 × 10 mm each, were chemically treated to immobilize on the sensor surface the anti-cortisol and anti-*E. coli* antibodies, respectively. This procedure was adapted from [31,34] and is schematically depicted in Figure 2. Therefore, the first step consisted of hydroxylating the glass slide’s surface with a “piranha” solution (3:1 volume ratio of H_2_SO_4_:H_2_O_2_) for 10 min, followed by thorough rinsing with DI water. Subsequently, amination was carried out by incubating the samples for 1 h in a (70:30% *v*/*v*) ethanol–DI water mixture containing 5% *v*/*v* APTES and 2% *v*/*v* DMOAP (which induces a homeotropic alignment of the LC molecules). Then, the glass surfaces were thoroughly rinsed with DI water to eliminate unbound molecules. Subsequently, the glass slides were subjected to 120 °C for a 20 min cure treatment. The third step consisted of incubating for 2 h the amine-terminated glass slides in a solution of anti-cortisol (100 µg/mL) or anti-*E. coli* (100 µg/mL) antibodies with EDC/NHS (0.2 M/0.5 M) in PBS, followed by thorough washings with PBS. The antibody concentration of 100 µg/mL was chosen based on a prior concentration-optimization study conducted in our previous work [31]. The EDC/NHS chemistry enables the efficient and specific activation of the carboxyl groups on the Fc region of the antibody, allowing them to bind covalently to the amine-terminated sensor surface. For surface passivation, the glass slides were incubated for 2 h in a BSA solution containing 10 µg/mL. This procedure ensures the blocking of unreacted sites after antibody immobilization, preventing nonspecific binding. Finally, the surfaces were thoroughly rinsed and incubated in PBS overnight before performing the detection tests.

Following the tests, the glass substrates were paired and glued at the edges using an epoxy adhesive. A drop of approximately 3 µL of LC was then introduced between the slides via capillary action, filling the cell. The resulting sensor presented an average thickness of 1.5 µm between the two glass surfaces since several measurements were performed across different sensors. The schematic of the developed immunosensor is also depicted in Figure 2. The right panel of Figure 2 illustrates the binding of the analyte to the specific antibody, thereby distorting the homeotropic alignment of the liquid crystal molecules. As a result, the incident light, polarized by the first polarizer, undergoes polarization rotation in the LC medium and emerges after passing through the second polarizer (crossed regarding the first polarizer), resulting in a colorful image that indicates a positive detection, as the analyte is detected. On the contrary, regarding the left panel, the orientation of the LC molecules is not disrupted, resulting in a black image due to the extinction of the incident light by the second polarizer, as light does not undergo polarization rotation in the LC medium. This result indicates a negative detection, as no analyte binds to the antibody.

The immunosensors were examined using a POM, specifically an Olympus BX51 microscope equipped with linear polarizers, a CCD camera (Olympus DP73), and Stream Basic v1.9 software from Olympus (Olympus, Hamburg, Germany). The sensors were positioned between crossed linear polarizers, and images were captured in transmission mode using a 10× objective lens (Olympus MPlanFL N, Olympus, Hamburg, Germany). Image scaling was performed automatically by the software, with the scale bar in each image representing 100 µm. Illumination was provided by a halogen lamp (KL 2500 LCD, SCHOTT). To quantify the transmitted light through each immunosensor, the transmitted light spectra were recorded using the integrated monochromator from the Sense+ system (Sarspec) in combination with LightScan 2.0 software. The integrated spectral light flux was obtained by integrating the spectral curve over the wavelength range of 300 to 1000 nm. Under identical conditions, each data point represents the mean of three independent measurements performed on three immunosensors, except for tests conducted at the industrial unit for *E. coli* detection, where two independent measurements were conducted on two immunosensors. The error bars represent the largest standard deviation.

### 2.3. Measuring Protocol: Cortisol Detection, Selectivity Test, and Real RAS Samples Testing

The detection tests for cortisol were conducted using five concentrations (0.1, 1, 5, 10, and 50 ng/mL) prepared in DI water. These concentrations were obtained through successive dilutions of a stock solution. The stock solution, at a concentration of 0.1 mg/mL, was prepared by dissolving cortisol in a solvent mixture of 1% ethanol and 99% DI water. The measurement procedure involved immersing the functionalized glass slides in each solution for 30 s, followed by washing with DI water.

Cortisol selectivity tests were conducted using testosterone, glucose, and cholesterol as control analytes. Following the functionalization procedure outlined in Section 2.2, sensors were prepared with anti-cortisol antibodies and tested with the following concentrations: 2.5 ng/mL and 20 ng/mL of testosterone, 6 ng/mL and 18 ng/mL of glucose, 10 ng/mL and 50 ng/mL of cholesterol, and 5 ng/mL and 50 ng/mL of cortisol.

To evaluate the performance of the developed LC-based immunosensors in a real scenario, tests were performed on samples collected from different zones of the RAS (with corvina) under normal conditions without stress induction. Therefore, the glass slides were functionalized with anti-cortisol antibodies following the procedure described in Section 2.2 and tested on three different samples. One water sample was collected directly from the fish tank, and the others were collected after passing through the biofilter and the ozone injection. Analytical extraction was performed through liquid chromatography–tandem mass spectrometry (LC-MS/MS) to quantify the real cortisol concentration in tanks with corvina, as presented in Table 1. The cortisol concentrations reported in Table 1 correspond to snapshot measurements obtained from samples collected from each RAS zone. For each RAS zone, three independent 50 mL water samples were collected and analyzed at the same time point under stable operating conditions without stress induction. For each RAS zone, all three water replicates (50 mL each) yielded the same analytical response. The results show that samples collected from the part of the tank with an ozone injection and a biofilter present a cortisol concentration substantially lower than that of samples collected directly in contact with fish. This may be related to the capacity of these filters to gradually reduce the cortisol levels in the tanks’ water [15]. Thus, the sample collected directly from the fish tank must present a higher concentration of cortisol, which decreases after passing through the filters and is lower after the ozone injection.

### 2.4. E. coli Detection Tests in an Industrial Unit

**Figure 3:**
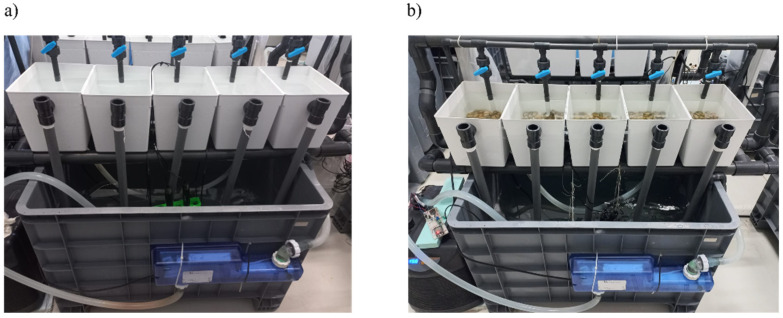
Industrial unit tanks with (**a**) contaminated water and (**b**) contaminated clams.

LC-based immunosensors for *E. coli* detection developed and tested in previous work [31] were tested on an industrial unit to assess the concentration of *E. coli* in tanks with contaminated water and clams (*Venerupis corrugata*), as shown in Figure 3, over a depuration of 24 h and 48 h, respectively. Depuration consists of eliminating bacteria using UV radiation with a device positioned in the water tank circuit.

In the first case, the water in the tank without bivalve mollusks was purposely contaminated with a high concentration of *E. coli,* and six water samples were collected throughout the 24 h depuration process. Thus, samples were obtained at the time of contamination (0 h), after 30 min, 3 h, 4.5 h, 22.5 h, and at the end of 24 h.

LC-based immunosensors were also used to assess the concentration of *E. coli* in contaminated clams throughout a 48 h depuration process. This time, clams were taken from three different tanks at 0 h and after 24 h, 42 h, and 48 h, and crushed to obtain a homogenized sample. This procedure was carried out in three different tanks. For the depuration in tank 1, three UV LEDs in a circle were used; in tank 2, three UV LEDs in a line were used; and in tank 3, a commercial UV lamp was used. The use of a commercial UV lamp in conventional depuration systems is typically associated with higher power consumption. For this reason, two alternative UV LED configurations were tested to evaluate whether UV LEDs could achieve a similar depuration efficiency while significantly reducing energy requirements. Two different geometries were implemented: a circular arrangement, in which the LEDs were positioned around the tube to maximize irradiation coverage and ensure that a larger portion of the circulating water was directly exposed to UV light, and a linear arrangement, where the LEDs were aligned along the tube to provide a more directional and concentrated irradiation path. These two LED structures were compared with the commercial UV lamp to assess their microbial reduction performance and potential energy-efficiency advantages.

It is essential to note that the *E. coli* concentration was determined through culture-based methods to validate the sensor responses.

### 2.5. Prototype Development

**Figure 4:**
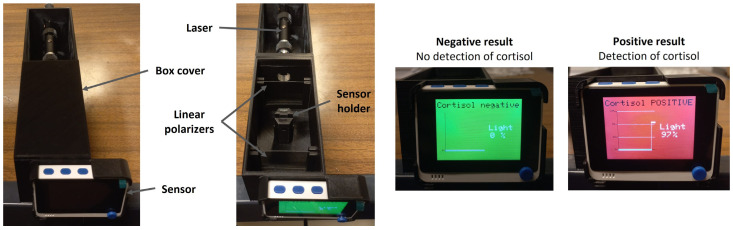
Prototype and respective possible results: positive (detection of cortisol) and negative (no detection of cortisol).

To enable the use of LC-based immunosensors in field-deployable and on-site applications, a portable, miniaturized, and user-friendly prototype was developed to replace POM analysis, as presented in Figure 4. Preliminary tests using a similar prototype were previously performed for *E. coli* immunosensors [35].

This prototype can show two possible results: a positive or a negative result, corresponding to the detection or non-detection of cortisol. The prototype is composed of a box divided into two parts. One part is designed for a laser, and the second part presents two crossed linear polarizers and a sample holder for the LC-based immunosensor. The laser beam, after passing through the polarizers and the immunosensor, reaches a photodetector that is incorporated in a WiO terminal (D51R), which displays the percentage of light that passes through the sensor, showing “Cortisol POSITIVE” if the percentage of light is equal to or higher than 50%, or “Cortisol negative” otherwise.

The LC-based immunosensors developed for cortisol detection, as described in Section 2.3, besides being analyzed with POM, were also used in the prototype for comparison. For the last case, the percentage of light that passes through each sensor was recorded.

## 3. Results and Discussion

### 3.1. Cortisol Detection

#### 3.1.1. Cortisol Binding Tests

**Figure 5:**
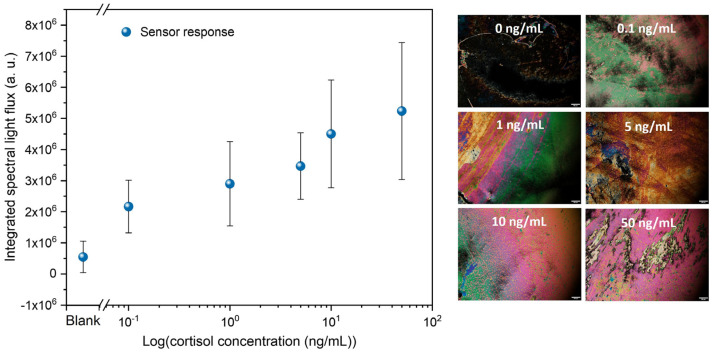
Integrated spectral light flux as a function of the cortisol concentration (0, 0.1, 1, 5, 10, and 50 ng/mL) in a semi-log scale. The scale bar in the POM images corresponds to 100 μm.

The LC-based immunosensors were immersed in cortisol solutions in DI water with concentrations ranging from 0.1 to 50 ng/mL to evaluate their performance. Therefore, the integrated spectral light flux for the five cortisol concentrations was analyzed in relation to the control sample, as shown in Figure 5. The POM images are representative of each of the tested immunosensors.

Figure 5 shows a logarithmic variation in the light flux with increasing cortisol concentration. This means that the higher the concentration, the more cortisol molecules are bound to the antibodies, which imposes distortion in the orientation of more LC molecules, resulting in a higher amount of light that emerges from the immunosensor. Accordingly, there is a positive relation between the number of cortisol molecules in the analyte and the distortion imposed on the LC molecule’s homeotropic alignment. Thus, there is an increase in light flux with the increase in cortisol concentration. Considering this set of results, a clear trend emerges in which higher cortisol concentrations correspond to higher light-flux values, even though some individual data points deviate slightly above or below the curve that would best fit this variation. From the POM images, this relation is evident, and the control sample (0 ng/mL) shows a negative result. The measured limit of detection (LOD) is 0.1 ng/mL, with an average light flux statistically distinguishable from that of the control sample; below this limit, cortisol is not detected, and the immunosensors appear completely dark. The LOD was determined based on a signal-to-noise (S/N) ratio of 3, defined as the lowest cortisol concentration producing a signal three times higher than the standard deviation of the blank (0 ng/mL). This choice reflects the intrinsic characteristics of LC-based optical immunosensors, whose response arises from spatially heterogeneous birefringence patterns and exhibits increased variability and a quasi-logarithmic behavior rather than a strictly linear response. Under these conditions, defining a robust linear slope is less reliable. In contrast, the S/N method provides a more appropriate and conservative criterion by identifying the lowest analyte concentration that yields a signal distinguishable from the background noise, thereby supporting proof-of-functionality at this stage of development.

Analyzing the two reported works on LC-based sensors for cortisol detection present in Table 2, our immunosensor presents a lower LOD value compared to the LOD reported in the more recent work by Saba Mostajabodavati et al. (0.414 ng/mL) for an LC aptasensor [33]. Although our attained LOD is higher than the LOD reported by Tsung-Keng Chang et al. [32], as demonstrated in the following section, our sensor exhibits high selectivity for cortisol, in contrast to the sensor developed by Tsung-Keng Chang et al., which lacks selectivity due to its ability to detect general surface interactions of biomolecules (e.g., BSA or cortisol). In this work, any molecule that disturbs the LC interface could generate a signal. In addition, the detection time is much longer. While these two approaches are primarily proof-of-concept and limited to single-analyte measurements under controlled laboratory conditions, as stated in the following sections, our work validates cortisol detection using real samples relevant to RAS, moving beyond buffer-based testing. Moreover, it demonstrates the integration of two fundamentally different immunoassays—cortisol (a small-molecule hormone) and *E. coli* (a whole-cell target)—using the same standardized LC sensing platform.

Overall, the LOD obtained with our sensor is lower than that reported in Table 2, except for two sensors; in those cases, the detection time is much longer. The detection time of our sensor (30 s) is among the fastest reported.

**Figure 6:**
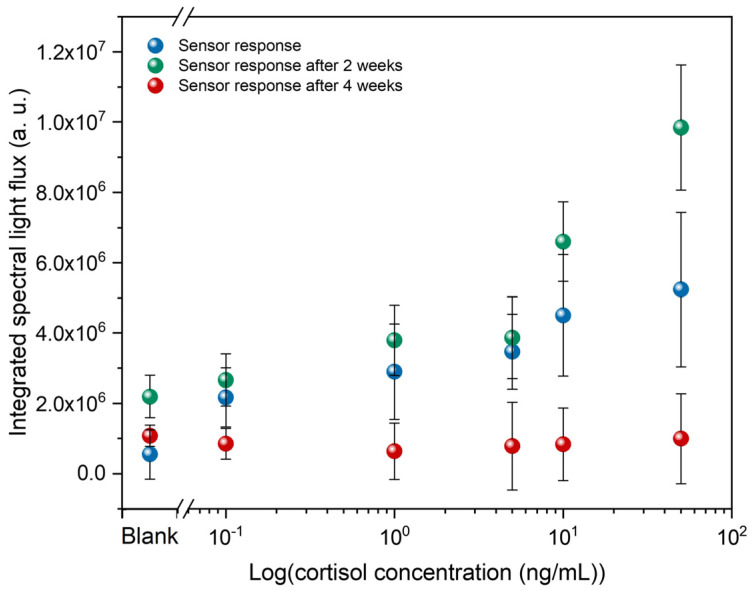
Temporal validity of LC-based immunosensors, including POM analysis after 2 and 4 weeks.

In addition, the temporal validity of these immunosensors was also evaluated. Thus, these immunosensors were analyzed in the POM at 2 and 4 weeks after their initial analysis. Figure 6 presents these results, showing that after 2 weeks, the light flux changes more noticeably at higher concentrations. After 4 weeks, the response of these immunosensors is entirely different from the initial analysis, showing virtually no change in light flux as cortisol concentration increases. This means that LC molecules are likely to reorganize over time until they align homeotropically. Therefore, the data suggest that the immunosensors exhibit a measurable response within the first 2 weeks after fabrication; however, significant temporal variations, especially at higher concentrations, indicate that signal stability remains a critical aspect to be further optimized.

The loss of reliability over time can be attributed to antibodies immobilized on the glass surface, which can gradually lose activity due to denaturation, desorption, or hydrolysis, reducing the number of available binding sites and weakening the LC alignment disruption. Also, the DMOAP-based silane layers that impose homeotropic LC alignment can undergo hydrolysis and subsequent structural reorganization/partial release over time when exposed to aqueous biological media, which compromises their long-term stability as homeotropic alignment layers. This phenomenon leads to diminished anchoring strength, causing the LC to revert to a uniform dark state regardless of analyte presence [42].

To prolong sensor stability, improvements may include optimizing storage conditions, reinforcing or replacing the silane alignment layer with more stable formulations, sealing and protecting the LC cell from moisture and evaporation, or storing functionalized slides separately and assembling the LC only at the time of use.

#### 3.1.2. Selectivity Test: Interfering Substances

**Figure 7:**
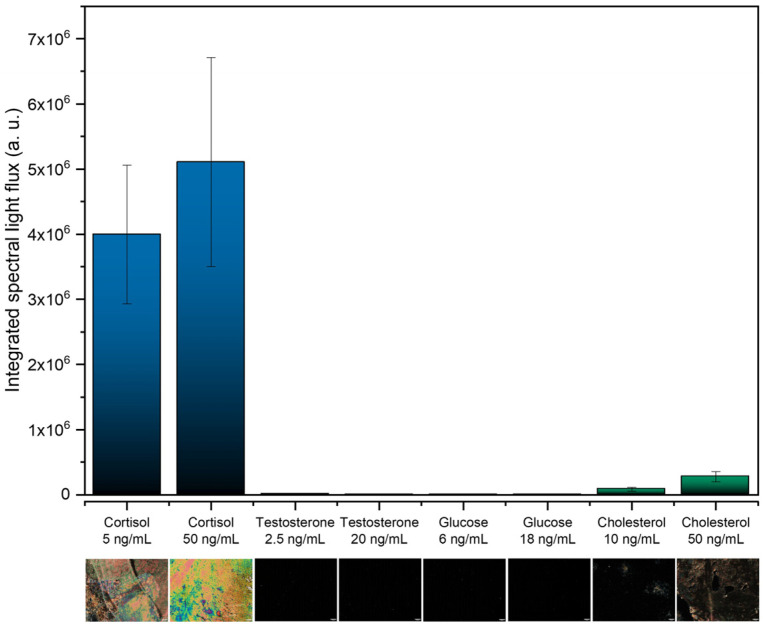
Selectivity test using testosterone, glucose, and cholesterol. The scale bar in the POM images corresponds to 100 μm.

The selectivity of the immunosensor in detecting cortisol is a crucial parameter to ensure that the presence of other analytes does not result in a false positive. In this way, to evaluate the selectivity, the developed LC-based immunosensors were also tested for possible interfering substances, including testosterone, glucose, and cholesterol. The integrated spectral light flux was analyzed and is shown in Figure 7, along with the corresponding POM images.

Positive results with colorful textures and a high light flux were obtained for both cortisol concentrations (5 and 50 ng/mL), whilst for the other immunosensors, exposed to the other analytes, negative results were obtained with dark POM images. These results demonstrate the specificity of this LC-based immunosensor for cortisol and its ability to suppress interference from other biomolecules.

#### 3.1.3. Test in Real RAS Samples

The LC-based immunosensor’s performance was evaluated in a real scenario using water samples collected from three different zones of the RAS, including directly from the fish tanks, the biofilter, and ozone injection.

**Figure 8:**
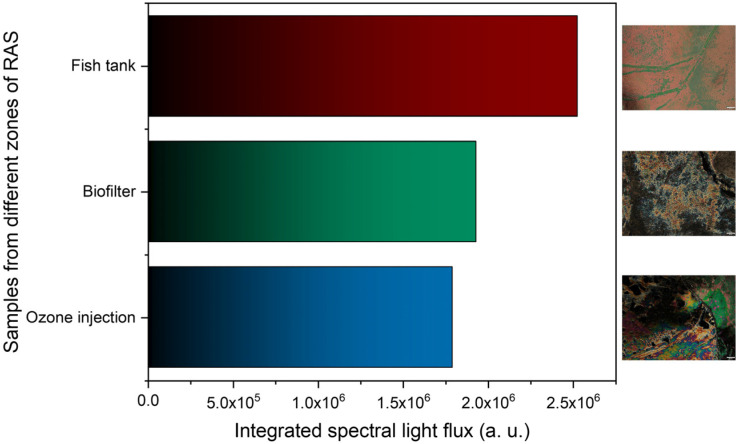
Test in a real scenario using water samples from three zones of the RAS. The scale bar in the POM images corresponds to 100 μm.

As described in Section 2.3, the water samples collected near both filters (ozone injection and biofilter) show cortisol concentrations substantially lower than those collected directly in contact with fish, with lower concentrations near the ozone injection. Observing Figure 8, which shows the results of the LC-based immunosensors tested on those three samples, it is possible to conclude that our sensors responded according to the concentrations shown in Table 1. The light flux attained for the fish tank was substantially greater than the other two cases, which were lower for the ozone injection. The LC-based immunosensor was able to detect cortisol in RAS water samples, even at lower or higher concentrations.

#### 3.1.4. Prototype Analysis

The ultimate goal is to have a portable, miniaturized, and user-friendly monitoring device capable of detecting cortisol for application in different fields. Therefore, a prototype that uses the LC-based immunosensors was developed.

**Figure 9:**
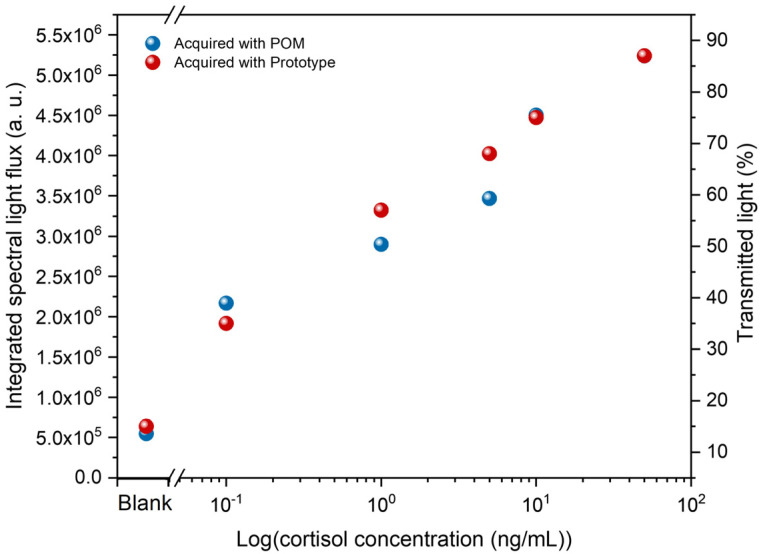
POM and prototype comparison sensor responses.

The same immunosensors tested in section A were analyzed using the novel prototype that rapidly measures the percentage of transmitted light through a specific sensor region, providing results within seconds. To evaluate the response of this prototype, a comparison between the prototype’s results and the POM analysis was performed, as presented in Figure 9. Each analysis shows an increase in light flux (POM analysis) and transmitted light (prototype analysis) with increasing cortisol concentration. There is only a slight difference between 1 ng/mL and 5 ng/mL, with the transmitted light slightly higher than the light flux. However, the overall responses were similar, suggesting that the prototype can consistently indicate the presence of cortisol.

### 3.2. E. coli Detection Tests in Real Samples

**Figure 10:**
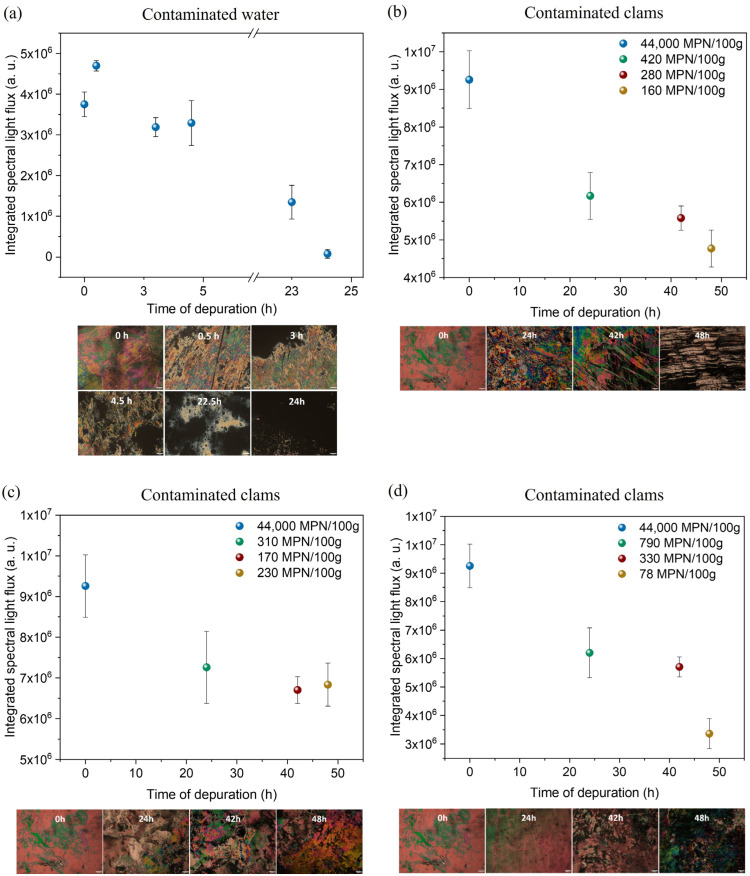
Results of the LC-based immunosensor tests in contaminated water and clam samples. Integrated spectral light flux values vs. depuration time and corresponding POM images for (**a**) tank with contaminated water, (**b**) Tank 1, (**c**) Tank 2, and (**d**) Tank 3. The scale bar in the POM images corresponds to 100 μm.

As the depuration process progresses, the concentration of *E. coli* should decrease, and the sample collected after 24 h should show a much lower concentration than the samples collected first. This is, in fact, supported by the results, in which the images at 0 h and 30 min are much more colorful than the image at 24 h. By calculating the integrated spectral light flux associated with each sample, it is possible to observe that there is a general tendency for this value to decrease over time (Figure 10a). The maximum value of 4.70 × 10^6^ a.u. decreased to 1.72 × 10^5^ a.u. for the 24 h sample. It is important to note that the maximum was not detected for the 0 h sample. However, this can be explained by the fact that the bacteria may not have been well distributed throughout the tank, so when the water began circulating, it increased the value of the sample collected. Regardless, as only 30 min had passed since the start, it was unlikely that a considerable reduction in *E. coli* would have happened.

As shown in Figure 10b–d, as the depuration process progresses in the three tanks, the light flux and the concentration of *E. coli* decrease, and the sample taken after 48 h shows much lower light flux and concentration than the samples collected at 0 h. Furthermore, there is a general tendency for the light flux value to decrease over time, showing compatible results between the light flux values and *E. coli* concentration values. Moreover, it is important to note a maximum concentration value of 4.4 × 10^4^ *E. coli* (MPN/100 g) at the beginning of the process, corresponding to a bivalve production area classified as C (bivalves can be harvested and are only intended for prolonged transposition or processing in an industrial unit). After 48 h, good results were achieved, reaching class A with 1.6 × 10^2^ *E. coli* (MPN/100 g) in tank 1 (UV LEDs in circle), 2.3 × 10^2^ *E. coli* (MPN/100 g) in tank 2 (UV LEDs in line), and 7.8 × 10^1^ *E. coli* (MPN/100 g) in tank 3 (commercial UV lamp). These results also indicate that both UV LED configurations promoted microbial reduction comparable to that achieved with the commercial UV lamp. Although the final *E. coli* concentration obtained with the commercial lamp was slightly lower, all three systems successfully reached class A levels after 48 h, meeting the regulatory requirements for bivalve commercialization and consumption. Moreover, among the LED-based approaches, the circular LED arrangement proved to be more efficient than the linear configuration, achieving a greater reduction in *E. coli*. These findings suggest that UV LEDs, particularly when arranged in a circular configuration, may be a viable, more energy-efficient alternative to traditional commercial UV lamps for industrial depuration processes.

Although this LC-based immunosensor still presents limitations in quantifying *E. coli*, it has potential for the intended application. Further testing is necessary to fully explore its detection and application limits.

These results demonstrate that the same LC transduction mechanism can reliably support both competitive and sandwich immunoassay formats, enabling sensitive detection of chemically and structurally distinct targets such as cortisol and *E. coli* cells within a unified sensing architecture. The ability of the LC sensor to maintain stable alignment and measurable optical responses across two fundamentally different immunoassay formats underscores the robustness of the LC–interface interactions.

## 4. Conclusions

This study demonstrates the successful development and application of LC-based immunosensors for the optical detection of cortisol and *E. coli* in different aquaculture settings. The immunosensors exhibited high sensitivity and selectivity, enabling real-time, label-free detection of these critical analytes. The results demonstrate that LC-based immunosensors are capable of detecting cortisol in RAS water samples, supporting their potential use for assessing stress-related variations in fish populations. Similarly, the immunosensors tracked *E. coli* contamination in water and bivalve mollusks throughout the depuration processes, providing a rapid and reliable alternative to traditional microbiological methods.

Furthermore, integrating these immunosensors into a user-friendly prototype highlights their potential for applications in aquaculture management. This portable device facilitates on-site monitoring, reducing the reliance on complex laboratory procedures. The findings suggest that LC-based immunosensors can play a crucial role in enhancing food safety, improving aquaculture productivity, and ensuring the well-being of aquatic species. Importantly, this work demonstrates the versatility of a single, standardized LC sensing platform to accommodate two fundamentally different immunoassay formats, targeting both a small molecule (cortisol) and pathogens (*E. coli*). This dual applicability highlights the potential of LC-based biosensors as a universal transduction strategy rather than an application-specific solution. In this context, the incorporation of microfluidic components into the LC platform presents a promising direction to achieve multiplexed analysis while reducing sample and reagent consumption. However, careful design of channel geometry and flow conditions will be essential to preserve LC alignment and maintain sensing performance. Future research should focus on optimizing the prototype and sensor fabrication, as well as on further validating the prototype across diverse aquaculture environments. Equally important is the future implementation of image-processing approaches supported by machine learning and artificial intelligence to enable more accurate, reproducible, and automated analysis of birefringence patterns, thereby minimizing operator dependence and ensuring reliable quantification of analytes such as *E. coli* and cortisol.

## Figures and Tables

**Table 1:** Cortisol concentration in the different RAS zones.

RAS Zones	Cortisol Concentration (ng/mL)
Fish tanks	4.22
Biofilter	3.06
Ozone injection	2.68

**Table 2:** Comparison of cortisol sensors in terms of LOD and detection time.

Type of Sensor	LOD (ng/mL)	Detection Time (min)	Reference
LC aptasensor	0.414	<8	[33]
LC marginally aligned sensor	0.003	~2	[32]
Amperometric electrochemical sensor	0.54	50	[36]
OECT and EIS sensor	0.036	5	[37]
MIP electrochemical sensor	2	-	[38]
3D origami microfluidic fluorescence aptasensor	6.76	25	[39]
Quantitative chemiluminescence-based LFIA integrated in a smartphone	0.3	-	[40]
MIP fluorescence polarization-based nanoplatform	0.28	-	[41]
LC immunosensor	0.1	0.5	Our work

OECT: organic electrochemical transistors; EIS: electrochemical impedance spectroscopy; MIP: molecularly imprinted polymers; LFIA: lateral flow immunoassay.

## Data Availability

The data presented in this study are available on request from the corresponding author.
